# A Smart, Textile-Driven, Soft Exosuit for Spinal Assistance

**DOI:** 10.3390/s23198329

**Published:** 2023-10-09

**Authors:** Kefan Zhu, Phuoc Thien Phan, Bibhu Sharma, James Davies, Mai Thanh Thai, Trung Thien Hoang, Chi Cong Nguyen, Adrienne Ji, Emanuele Nicotra, Hung Manh La, Tat Thang Vo-Doan, Hoang-Phuong Phan, Nigel H. Lovell, Thanh Nho Do

**Affiliations:** 1 Graduate School of Biomedical Engineering, Faculty of Engineering, UNSW Sydney, Kensington Campus, Sydney, NSW 2052, Australia; kefan.zhu@unsw.edu.au (K.Z.); bibhu.sharma@unsw.edu.au (B.S.); j.j.davies@student.unsw.edu.au (J.D.); maithanh.thai@unsw.edu.au (M.T.T.); trungthien.hoang@unsw.edu.au (T.T.H.); cong.c.nguyen@unsw.edu.au (C.C.N.); adrienne.ji@unsw.edu.au (A.J.); e.nicotra@unsw.edu.au (E.N.); n.lovell@unsw.edu.au (N.H.L.); 2 College of Engineering and Computer Science, VinUniversity, Hanoi 100000, Vietnam; 3 Advanced Robotics and Automation Lab, Computer Science and Engineering, University of Nevada, Reno, NV 89512, USA; hla@unr.edu; 4 School of Mechanical & Mining Engineering, The University of Queensland, St. Lucia, QLD 4072, Australia; t.vodoan@uq.edu.au; 5 School of Mechanical and Manufacturing Engineering, Faculty of Engineering, UNSW Sydney, Kensington Campus, Sydney, NSW 2052, Australia; hp.phan@unsw.edu.au; 6 Tyree Foundation Institute of Health Engineering (IHealthE), UNSW Sydney, Sydney, NSW 2052, Australia

**Keywords:** wearable device, artificial muscle, smart textile, soft exosuit, spinal assistance

## Abstract

Work-related musculoskeletal disorders (WMSDs) are often caused by repetitive lifting, making them a significant concern in occupational health. Although wearable assist devices have become the norm for mitigating the risk of back pain, most spinal assist devices still possess a partially rigid structure that impacts the user’s comfort and flexibility. This paper addresses this issue by presenting a smart textile-actuated spine assistance robotic exosuit (SARE), which can conform to the back seamlessly without impeding the user’s movement and is incredibly lightweight. To detect strain on the spine and to control the smart textile automatically, a soft knitting sensor that utilizes fluid pressure as a sensing element is used. Based on the soft knitting hydraulic sensor, the robotic exosuit can also feature the ability of monitoring and rectifying human posture. The SARE is validated experimentally with human subjects (N = 4). Through wearing the SARE in stoop lifting, the peak electromyography (EMG) signals of the lumbar erector spinae are reduced by 22.8% ± 12 for lifting 5 kg weights and 27.1% ± 14 in empty-handed conditions. Moreover, the integrated EMG decreased by 34.7% ± 11.8 for lifting 5 kg weights and 36% ± 13.3 in empty-handed conditions. In summary, the artificial muscle wearable device represents an anatomical solution to reduce the risk of muscle strain, metabolic energy cost and back pain associated with repetitive lifting tasks.

## 1. Introduction

Work-related musculoskeletal disorders (WMSDs) are still jeopardizing the health of workers. Within WMSDs, approximately 40% are estimated to be back-related [1]. Prolonged standing and repetitive handling have been established as significant risk factors for WMSDs. For example, in aviation, airport baggage handlers have lifting, carrying and holding tasks, which are known to induce lumbar disc herniation and lower back pain (LBP) [2,3,4]. Studies have shown that 62.7% of handlers have LBP [5]. Extensive research has demonstrated that back pain, particularly LBP, can substantially diminish the quality of life and impede the ability to carry out activities of daily living (ADL) [6,7,8,9]. Further, LBP incidence in baggage handlers increases significantly with time [10]. While squat lifting is preferable over stoop lifting, as it hinders the forward flexion of the spine, reducing the internal spinal loading [11], stoop lifting cannot be completely avoided. Similar to repetitive stoop lifting, prolonged and incorrect sitting postures, such as slouching to one side, supporting one’s chin with a hand, or resting on folded arms, have the potential to exacerbate musculoskeletal back pain and contribute to the development of spinal deformities [12,13]. This means that preventing WMSDs that are associated with stoop lifting and prolonged and incorrect sitting postures should receive significant priority. Furthermore, for spine-assistive devices, the benefits go beyond reducing the risk of back pain. They can help workers to increase productivity, assist patients with post-surgery rehabilitation and improve the ability of older adults to perform daily activities. On the other hand, sitting posture monitoring not only helps in the early detection and correction of abnormal posture, but also guides the education of individuals about maintaining a healthy posture, thereby preventing long-term complications.

Numerous active wearable devices, either pelvic-joint-assistive devices or spine-assistive devices, have been proposed. Pelvic-joint-assistive devices apply an additional moment on the pelvic joint rotation in the sagittal plane [14]. A pelvic-joint-assistive device developed in [15] utilizes two motors placed on either side of the hip joint to generate torque on the joint, thereby assisting in trunk flexion and extension. The device reduced approximately 34.0% of the average integrated electromyogram (iEMG) in the erector spinae during repetitive lifting. Another representative pelvic-joint-assistive device is hybrid assistive limb (HAL) lumbar support, which has been shown to reduce 14% of the myoelectric activity of the lumbar erector muscles [16]. Compared to HALs, another prototype of a lower-back robotic exoskeleton exhibited a reduction of approximately 34% in the erector spine muscles [17]. However, such rigid exoskeletons typically exhibit a considerable weight, thereby introducing additional mass and inertia at the distal segments of the user’s body. These factors can burden the metabolic cost of the user’s movements [18]. Furthermore, the misalignment of joints is one inevitable problem that is evident with rigid wearable devices, which may compromise the original physiological exercise by producing unsatisfactory interaction forces on the human limbs [18].

Spine-assistive devices apply a compression force, which acts in parallel with the back muscles such as the lumbar erector spinae (LES) or latissimus dorsi (LD) to assist their function [14]. The assistive performance of these devices can reduce the electrical activity of the lower back muscles by between 10% and 40% [19,20,21]. For example, a cable-driven soft spine wearable device consisting of a hyper-redundant continuum mechanism was developed to mimic the compression force of the erector spinae (ES) muscles, which was observed to reduce LES usage by 30% [22]. Another soft power suit utilizes two twisted string actuators (TSAs) fixed at the lower back, reducing the muscle activation required for dynamic lifting by 21.4–25.2% [23]. However, these actuators are associated with high friction loss, low speed, and high nonlinear hysteresis, making them challenging to control. Additionally, the device still contains rigid structures, which do not interface well with the human body, reducing the comfort and portability of the devices [24]. An examination of 52 wearable devices revealed that a mere 11% of them were classified as fully soft [22,23,25,26,27]. Within the realm of fully soft wearable devices, flexible textile materials stand as the prevalent solution for ensuring a compliant connection with the user’s body [28,29,30]. These materials serve a dual purpose: they facilitate the transfer of assisting forces and maintain the suit in an optimal configuration, thereby enhancing compatibility with ADL and ensuring safety [30]. Regarding passive devices, they are found to be better suited for light assistance tasks with limited dynamic movements. An analysis involving a passive personal lift assistive device demonstrated that the decrement in metabolic cost for lifting was not significant [31]. Moreover, as stoop lifting is highly dynamic and demands a considerable joint load, it would necessitate the use of more sophisticated active solutions [32].

Furthermore, numerous sitting-posture-monitoring devices have been developed. Most posture-monitoring devices can be divided into inertial measurement unit (IMU) devices and the incorporation of sensors [33,34] into smart textiles or haptics [35,36,37,38,39]. Puranik et al. developed a wearable device for posture monitoring. The device works primarily through the use of a flex sensor and an IMU in the waist subsystem [40]. The resistance of the flex sensor varies based on the bending of the back, and a microcontroller can detect this signal and vibration feedback is provided for a user. However, the monitoring function of the device is limited to the sagittal plane and curvature of the lumbar spine. Differences in sitting posture cannot be detected. For the incorporation of sensors into smart textiles, Jiang et al. developed a knitted self-powered sitting position monitoring vest for assisted sitting posture monitoring and correction [41]. The device is knitted with a conductive fibre (10% strain) and nylon yarn. Sensor arrays are integrated into various regions of the garment and are employed to ascertain the posture of subjects. One limitation is the increase in estimation errors that can occur because of the relative movement of smart textiles against the body [42]. The significant drawbacks of these devices mean that they are incapable of providing the correct posture for individuals, nor do they provide guidance on achieving proper alignment, because they lack an actuator mechanism to autonomously adjust and correct the sitting posture of individuals. In addition, nano soft sensors have been widely used in human motion recognition with triboelectric nanogenerators [43,44,45]. Yang et al. developed an ion gel fibrous membrane for physiological detection and monitoring with electrospinning technology [46]. This capacitive sensor displays high sensitivity, repeatability and durability. However, the strain is limited below 3.6%, which is not satisfactory for human back posture monitoring. Another graphene textile triboelectric nanogenerator was developed by Yao et al. [47]. The device was constructed using scalable conductive graphene yarns, allowing it to stretch up to 150%, making it ideal for wearable applications. Additionally, it boasts high durability and can adapt to a wide range of complex deformations. The device is designed to identify four specific types of human movements. However, it lacks the capability to analyse the nature of the deformation or its axis, despite its adaptability to various deformations. While experiments primarily centred on joint bending, monitoring the spine’s posture proved to be challenging due to its similarity to spine bending and twisting.

**Figure 1:**
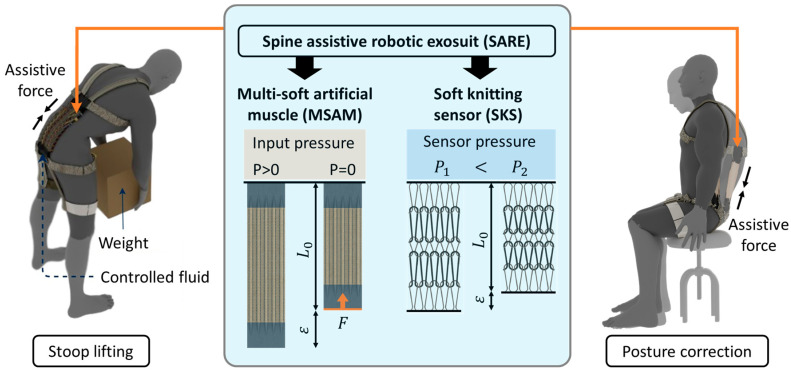
Overview of the spine-assistive system for stoop lifting tasks and rectifying sitting postures based on multi-soft artificial muscle (MSAM) and soft knitting sensors (SKSs).

To address the aforementioned issues through active assistance, this study develops a soft and lightweight spine-assistive robotic exosuit, which is composed of a knitting sensor, an artificial muscle actuator and fabric. The device can provide useful assistance during repetitive lifting tasks to reduce the risk of back-related musculoskeletal disorders. Concurrently, it introduces a novel concept to address and resolve posture monitoring issues, thereby offering a comprehensive solution to both assistive and preventive care in the domain of spinal health (Figure 1).

## 2. Materials and Methods

### 2.1. Mechanical Design

The spine-assistive robotic exosuit (SARE) comprises two essential components integrated into a smart textile—the soft knitting sensor (SKS) and the multi-soft artificial muscles (MSAMs). The SKS is a highly conformable sensor that uses a hydraulic pressure-based mechanism to detect strains and curvatures of the spine. The SKS is fabricated with a knitting technique to increase the longitudinal sensitivity. When attached to the human back, the length of the SKS adjusts passively with the curvature of the spine during lifting activities. As the curvature increases, the inner hydraulic pressure decreases, and this change is detected by the SKS, thereby informing the system of the spinal curvature in real-time.

**Figure 2:**
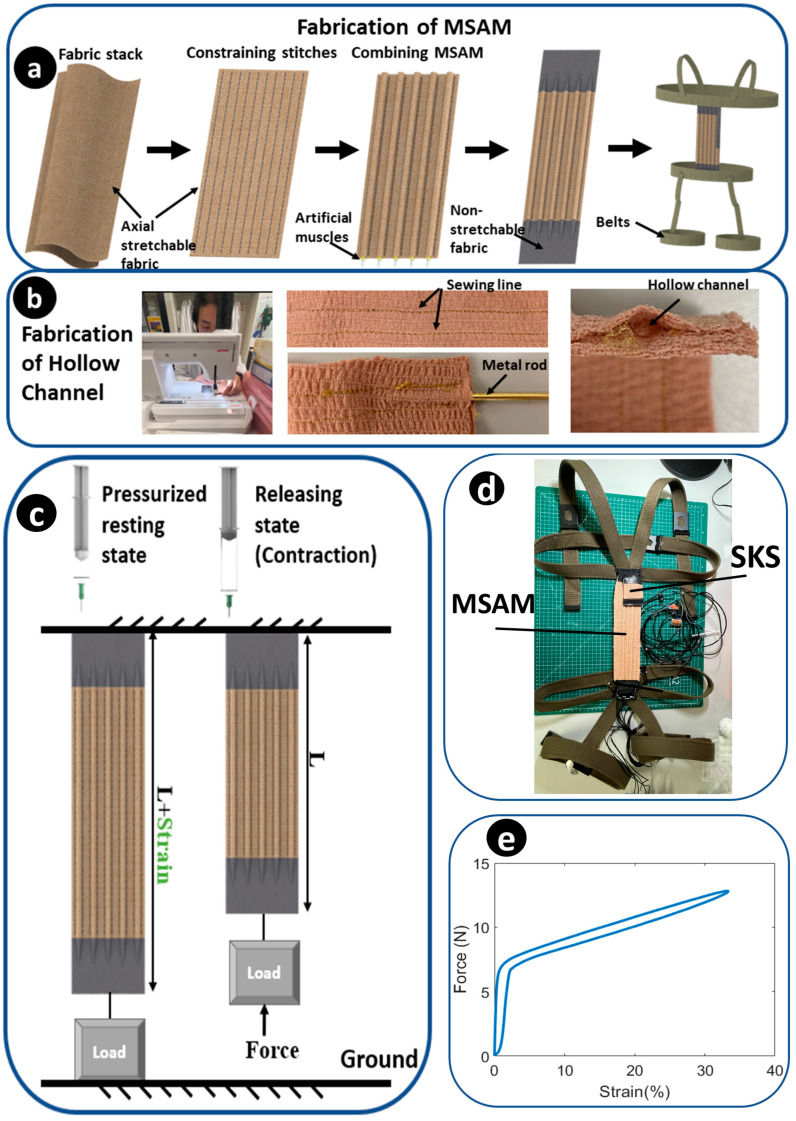
Fabrication and prototype. (**a**) Overview of fabrication of the MSAM. (**b**) Steps for fabricating the hollow channel with a sewing machine and metal rod. (**c**) Working principles of the MSAM. (**d**) Prototype of the SARE. (**e**) The compression force–strain profile for an individual artificial muscle.

The MSAM, on the other hand, acts as the active component of the SARE. Constructed from soft microtubules (NuSil™ Technology LLC, Carpinteria, CA, USA) and helical coils (McMaster-Carr Supply Co., Elmhurst, IL, USA), it is based on hydraulic filament artificial muscles [48]. It offers virtually infinite DOFs at optimum weight. A plastic guide tube is connected between a Luer-lock™ 3 mL syringe (BD, Franklin Lakes, NJ, USA) and the artificial muscle for transmitting hydraulic pressure to the artificial muscle. The working principles of the MSAM are as follows: in its resting state, the MSAM is elongated due to the pressurization achieved by pushing the syringe. For generating compressive force, releasing the piston of syringe, the stored potential energy within the microtubules and helical coils triggers the contraction of the MSAM, thereby lifting the load (Figure 2c). It mimics the function of the human erector spinae muscles. The durability and repeatability of the artificial muscles have been tested with the same structure, which can hold over 1000 cycles [29]. For 2000 s 0.1 Hz elongation experiments, the force is reduced by 11.9% and elongation shrinks by only 1.9% [48].

There are five fabrication process steps that utilize the MSAM (Figure 2a). Step 1: preparing two layers of axially stretchable fabrics that fit together. This stretchable fabric is chosen as a commercial self-adhesive compression bandage (3M, North Ryde, Australia), which is lightweight, porous and comfortable for patients. The metal structure of the MSAM is fully covered inside the fabric without contacting human skin. Step 2: hollow channels are made for inserting artificial muscles for combining the artificial muscles with the compression bandage using a zigzag stitch. The hollow channels offer a distinct advantage by effectively limiting the radial strain arising from the torsional forces that are exerted by the helical coil. The boundary of the hollow channel is created with a stitched line, produced with a sewing machine. A metal rod is inserted between two stitched lines to expand the hollow space (Figure 2b). This facilitates the easier passage of the artificial muscles through the channels. Step 3: after the hollow channels are completed, each artificial muscle is individually threaded through its respective hollow channel and aligned in parallel. Step 4: non-stretchable fabric is sewn to the two ends of the stretchable fabric to fixate the artificial muscles. Step 5: linking the MSAM to wearable belts, which are made of a comfortable nylon material. The upper portion is wrapped around the chest and passes over the shoulders to ensure secure fastening. The lower portion is wrapped around the pelvic and thigh regions (Figure 2d). The belts are adaptable to user dimensions using adjustable buckles. During lifting in a stooped position, the MSAM extends parallel to the spine, while the SARE pivots firmly on the distal and proximal supports.

### 2.2. Mathematical Modelling of the Musculoskeletal System

Taking inspiration from the force transmission within the muscles of the human back informs the development of an active fabric structure that is both comfortable and efficient in its design [23]. The ES is an essential structure for maintaining the upright posture of the human body, which includes the iliocostalis, longissimus and spinalis. It is responsible for the extension of the trunk and stabilizing the lumbar spine. From a thoracolumbar finite element (FE) model for lumbar posture, the maximum axial strain of the spine was estimated at 20% during stoop lifting tasks [49]. Also, the total predicted force on the LES was 242 N for a 45° flexion bending of the hip [50].

The mathematical modelling of a musculoskeletal system during lifting tasks can be intricate due to the numerous joints and muscles involved. While the FE method can provide numerical solutions [49], achieving convergence can be challenging due to the multitude of variables involved. However, a linked-segment model, such as in [51], can be used to simplify the human limb to minimize the degrees of freedom (DOFs). For the stoop lifting task, this paper considers a 2-DOF linked model, which has two joints: the hip and L5-S1 joint. The angular displacement of the lumbar-sacral joint represents the lordosis/kyphosis of the spine.

**Figure 3:**
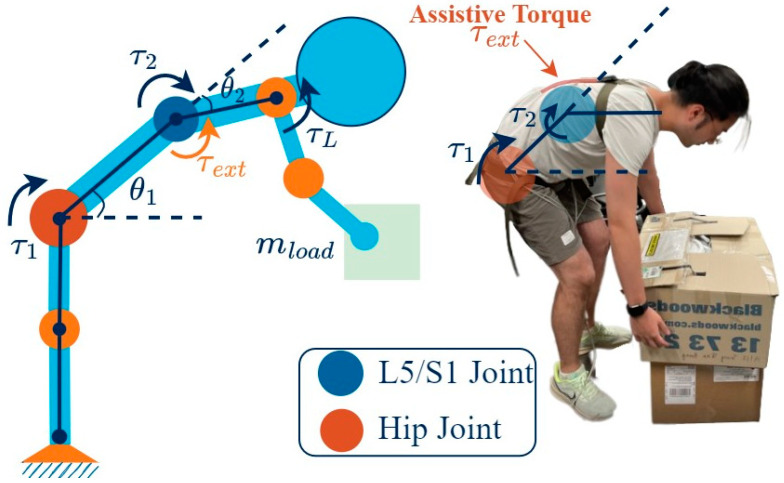
Modelling of stoop lifting, utilizing a linked segment model of the human musculoskeletal system.

Consider the generalized coordinate, $\theta \;\epsilon \;R^{2}$, as shown in Figure 3, actuated by joint torques, $\tau \;\epsilon \;R^{2}$. When lifting a load of mass ($m_{load}$), the moment created at the elbow joint is $\tau_{L}$. The assistance provided by the spine assistance device can be inferred as a torque, $\tau_{ext}$. The Lagrangian formulation, which involves the evaluation of kinetic function and potential function, was used to construct a canonical relation between kinetic and kinematic parameters as

(1) $$M\left(\theta \right)\ddot{\theta }+C\left(\theta ,\dot{\theta }\right)\dot{\theta }+G\left(\theta \right)=\tau +J{\left(\theta \right)}^{T}\tau_{L}-J_{2}{\left(\theta \right)}^{T}\tau_{ext}$$

**Figure 4:**
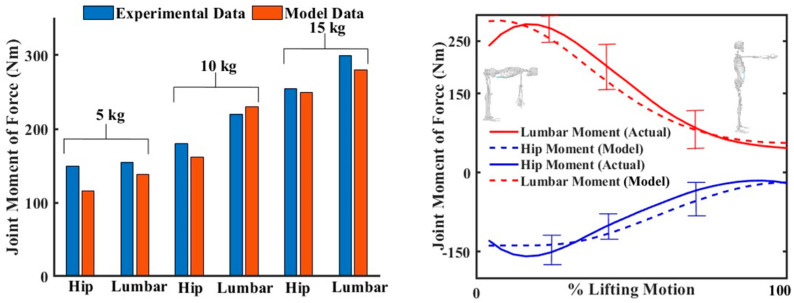
Modelling of stoop lifting, utilizing a linked segment model of the human musculoskeletal system. Comparison of a developed mathematical model with experimental data on stoop lifting from [50]. The (**left**) figure shows the peak hip torque and lumbar torque during stoop lifting of various weights. The (**right**) figure compares the hip torque and lumbar torque between actual and model data.

Here, the Jacobian $J\left(\theta \right)$ describes the relationship between the end-effector forces and the joint torques, and $J_{2}\left(\theta \right)$ is the Jacobian that describes the relation between the assistive force, $\tau_{ext}$, and the joint torques. While $J\left(\theta \right)$ is derived using the velocity relation between the end-effector and the joint, $J_{2}\left(\theta \right)$ is simply $J_{2}\left(\theta \right)=\left[0\;1\right]$, as assistive force is assumed to be applied only on the L5-S1 joint. M(θ) and $C(\theta ,\dot{\theta })$ are the mass matrix and Coriolis matrix, which depend upon the biomechanical parameters of the human subject. $G\left(\theta \right)$ is the gravity component. These equations are essential for determining the kinetic parameters that inform the design process, and are also critical for the development of functional control systems. The validation of the model was conducted by comparing experimental data from [52] (Figure 4) and the biomechanical parameters that are required in Equation (1), also derived from [52].

### 2.3. Actuation Mechanism

Based on the anatomy and developed mathematical models, an actuation mechanism was designed. The composite of the MSAM comprises 126 N/m spring constant helical coils and soft microtubules (E = 1.2556 MPa), and related steel tubes, guide tubes and syringes, in order to provide a compression force that reduces the LES force by 20%, which is 50 N. The output force generated by the artificial muscle is determined in [48] as

(2) $$F_{out}=\alpha \;EA_{t}\left(1-\frac{1}{1+\frac{x}{l_{i}}}\right)+k_{c}x$$

where α is the pre-sketched strain of the microtubule for inserting into the helical coil, *E* is the Young’s modulus for the soft microtubules, $A_{t}$ is the cross-sectional area of the soft microtubules, $k_{c}$ is the spring constant of the helical coil, x is the elongation value and $l_{i}$ is the initial length of the artificial muscle. The MSAM is designed to contain five artificial muscles. For a 20% MSAM strain, a 2 mL volume of liquid from a 3 mL standard syringe (Livingstone International Pty. Limited, Mascot NSW, Australia) with a 55.4 mm^2^ piston area is used. The thickness of each layer is 5 mm, which can be concealed underneath the garments completely. The weight of the MSAM was 56.8 g. The MSAM can apply 35 N over the entire 60 mm stroke range and a maximum of 50 N at a 360 mm length (Figure 2e).

To accurately control the strain of the MSAM, a linear actuator was employed and connected to the piston of the Luer-lock™ 3 mL syringe (BD, USA). The stroke of the linear actuator was controlled to alter the hydraulic pressure, thus realizing the desired strain of the artificial muscle. To enhance portability and comfort, the actuator and wearable components were separated. In order to determine an appropriate actuator, it is imperative to meet the theoretical requirements for thrust. The calculation of thrust involves both the internal pressure of the syringe and the pressure area of the piston.

Using Equation (3) [48], the internal pressure was determined to be 3.81 MPa, leading to a requirement of 1055.2 N of thrust to support the MSAM. As a result, the LACT8-1000BPL Linear actuator (Concentric™, Des Moines, IA, USA) was selected.

(3) $$P=F_{out}{\left(\frac{\pi }{4}d_{o}^{2}-\frac{\alpha A_{t}}{1+x/l_{i}}\right)}^{-1}$$

where *d*_0_ is the outer diameter of the microtubules.

### 2.4. Soft Knitting Sensor

#### 2.4.1. Overview and Working Principle

**Figure 5:**
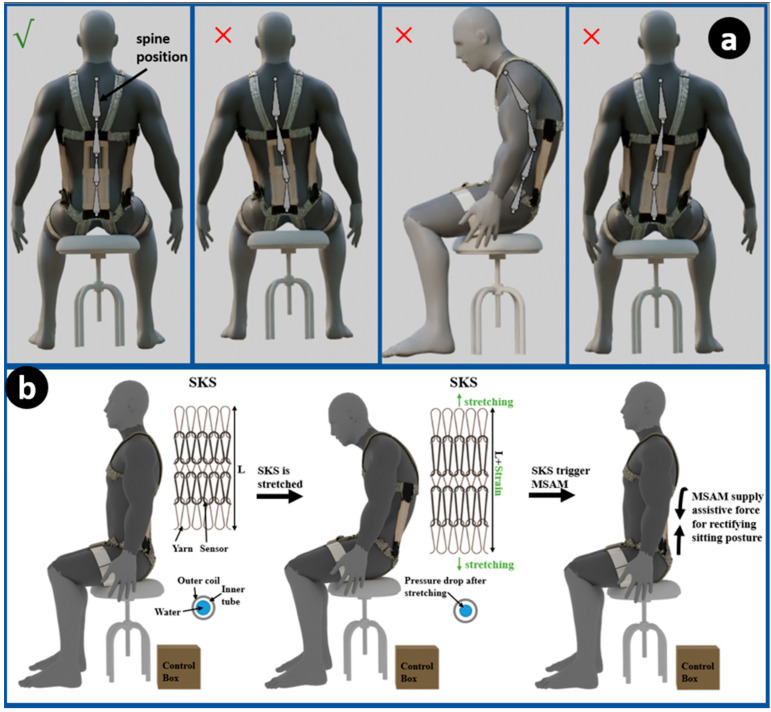
Posture correction methodology. (**a**) Schematic diagram showing the samples of different sitting positions that the SKS can detect. (**b**) Overview of the working principle of the SKS.

The soft knitting sensor (SKS) presented in this study draws inspiration from the hydraulic soft filament sensor and employs a knitting technique, which is capable of detecting changes in inner hydraulic pressure due to strain, informing the curvature of the spine from the measurement of hydraulic pressure. The SKS is attached to the MSAM. Because the MSAM shares the curvature and strain with the spine, it is stretched with the curvature of the MSAM during stoop lifting, causing the inner hydraulic pressure to drop with an increased curvature. By examining the relationship between the pressure and strain of the SKS, the curvature of the spine can be determined from the inner pressure signal. Figure 5b illustrates this relationship, displaying the working principle and patterns that emerge as the SKS reacts to variations in spinal curvature. The knitting technique offers significant advantages of high conformability and an interconnected structure. The knitted fabrics allow them to conform, making them well suited for applications involving direct contact with the human body. The interconnected structure allows for enhanced sensitivity by dividing the sensor into multiple short elements, enabling the precise interpretation of subtle changes in deformation or pressure.

To employ the SKS for the posture monitoring concept, the sensor is strategically integrated into wearable devices or garments, positioned along key regions of the body such as the sides and middle of the spine. As the individual assumes three different postures (Figure 5a), the sensor undergoes deformation proportional to the changes in spinal alignment. The hydraulic soft filament within the sensor is affected by this deformation, leading to alterations in hydraulic pressure. These pressure changes of three SKSs are then accurately measured and interpreted, providing real-time feedback on posture. By establishing a correlation between pressure variations and specific postural deviations, the SKS enables continuous posture monitoring. The sensor’s ability to capture even subtle changes in spinal curvature enhances its precision in detecting poor posture habits and correcting them with the MSAM. For example, the SKS can effectively detect instances of sitting with a slumped posture to one side, accompanied by a bent spine, due to its design and working principle. As an individual assumes a seated posture that is characterized by a pronounced slumping to one side, the SKS positioned on the contralateral side registers elongation—an indicative response to the compromised posture. This deformation-driven elongation leads to discernible shifts in the hydraulic pressure levels within the SKS’s internal structure. For instance, as depicted in Figure 5a’s second figure, when a volunteer slouches to the left side, the right side SKS will be stretched, resulting in a decrease in inner pressure, while the inner pressure of the left side SKS remains unchanged. Then, the pressure change in the SKS is detected and sent to a control system. Finally, the MSAM is triggered to activate force and rectify the postural misalignment.

#### 2.4.2. Design and Fabrication

**Figure 6:**
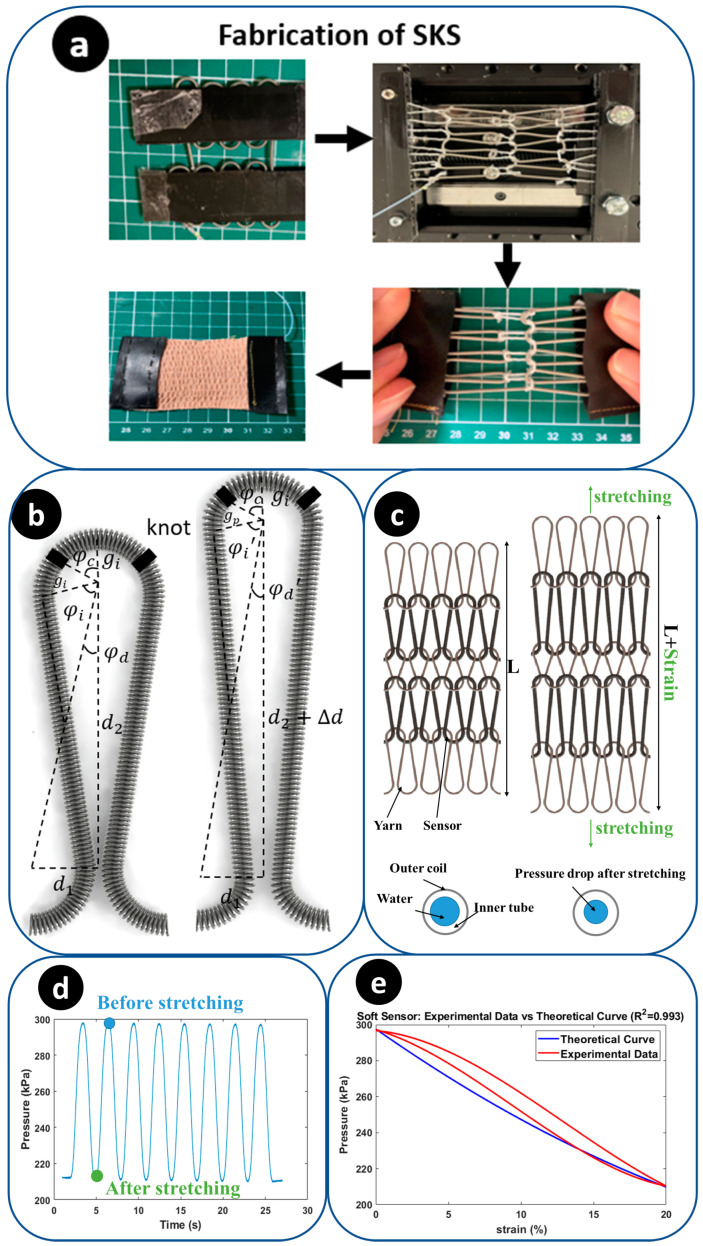
Sensing system along with the control system. (**a**) Overview of the SKS. (**b**) The left side is the parameter distribution of mathematical models. (**c**) Working principle of the soft sensor. (**d**) The pressure signal of the SKS changes during an 8-turn sine wave stretching. (**e**) The pressure–strain profile of the SKS under experimental data and theoretical data (R^2^ = 0.993).

The development of the SKS is achieved through the integration of a 600 mm length, 1.5 mm diameter helical coil (McMaster-Carr Supply Co., Elmhurst, IL, USA), and a 600 mm length, 1.2 mm diameter soft microtubule (NuSil™, Carpinteria, CA, USA). For the knitting technique, the sensor is bent to fit into a pre-designed pattern of two knitting tools. Subsequently, each loop is knotted using the same loop-shaped yarn, and the other side of the yarn is connected to another layer loop. Consequently, the sensor can be stretched by pulling on the two ends of the yarn. A total sensor length of 600 mm is knitted to obtain a 50 mm SKS. The SKS has a total of 5 loops for applying a high sensitivity. Finally, an axial stretchable fabric was sewn with a sewing machine to the surface of the sensors to limit the radial strain, and a non-stretchable fabric was combined with the two ends of the yarn to fixate the SKS. The fabrication of the SKS is shown in Figure 6a. The initial inner pressure of the SKS is 300 kPa, with a drop in pressure of 90 kPa as elongation reaches a 20% strain. The SKS demonstrates a sensitivity of 4.50 kPa/%, and the threshold is set at 280 kPa to prevent the inadvertent activation of the linear actuator. For the test, the SKS is fixed on the platform of a linear actuator. The linear actuator provides a sine wave movement function to elongate the SKS. The pressure change results are shown in Figure 6c.

#### 2.4.3. Mathematical Model and Characterization

To develop a mathematical model for the SKS, certain assumptions are made. Firstly, the silicone microtubule is considered to be a linearly elastic material under conditions of 300 kPa pressure and 20% strain. Secondly, the vertical distance between the two centres of the semicircle ($d_{2}$) increases linearly. Thirdly, the helical coil used in the sensor constrains the radial expansion of the microtubule. The SKS provides measurements of four parameters: the horizontal distance between two centres of the semicircle ($d_{1}$ and $d_{2})$, the radius of semicircle ($g_{i}$) and the angle between the knot position and the vertical vector ($\varphi_{c}$). After the sensor is stretched, two main variables that need consideration are the changed radius of the semicircle ($g_{p}$) and the difference in vertical distance between two centres of a semicircle after stretching (∆d). Geometrically, $g_{p}$ and ∆d can both be determined by following equations with respect to strain.

(4) $$∆d=strain\times (2g_{i}\times \cos\left(\varphi_{c})+d_{2}\right)$$

(5) $$∆d=2\sqrt{{\left(strain+1\right)}^{2}\times \left(\frac{d_{1}^{2}+d_{2}^{2}}{4}-g_{i}^{2}\right)-\frac{d_{1}^{2}+d_{2}^{2}}{4}+g_{p}^{2}}$$

Based on Barlow’s formula,

(6) $$P_{B}=\frac{2TS}{D}$$

where *P_B_* is the inner pressure of tube, *T* is the thickness of the tube wall, *S* is the allowable stress of the tube and *D* is the outside diameter of the tube. The relationship between the initial inner pressure and the changed inner pressure can be determined using Equation (6). Then, combined with Equations (4) and (5), the inner pressure changes of the SKS $\left(P^{\prime }\right)$ can be defined with one variable strain.

(7) $$\frac{P}{P^{\prime }}=\frac{\pi \times g_{p}\times \frac{180-\varphi_{d}\prime -\varphi_{c}-\varphi_{i}\prime }{180}+\sqrt{\frac{d_{1}^{2}+{\left(d_{2}+∆d\right)}^{2}}{4}-g_{p}^{2}}+\frac{2g_{i}\times \pi \times \varphi_{c}}{180}}{\pi \times g_{i}\times \frac{180-\varphi_{d}-\varphi_{c}-\varphi_{i}}{180}+\sqrt{\frac{d_{1}^{2}+d_{2}^{2}}{4}-g_{i}^{2}}+\frac{2g_{i}\times \pi \times \varphi_{c}}{180}}$$

where $\varphi_{i}{,\varphi }_{d},\varphi_{i}\prime ,\varphi_{d}\prime$ are the angles in the geometrical model for calculation (Figure 6b). *P* is the initial inner pressure of the sensor. The pressure–strain profile of the SKS under experimental data and theoretical data is shown in Figure 6d. The observed cubic curve characteristic in the experimental data can be seen as a combination of the delay of the SKS and a quadratic curve (theoretical data) The delay of the SKS could be attributed to the pre-stretching of the silicone tube, the slight slippage of knots within the yarn during the fabrication process or the sensor may not have been fully stretched initially at 0% strain.

## 3. Experimental Method

### 3.1. Lifting Assistance

#### 3.1.1. Surface Electromyography (sEMG)

**Figure 7:**
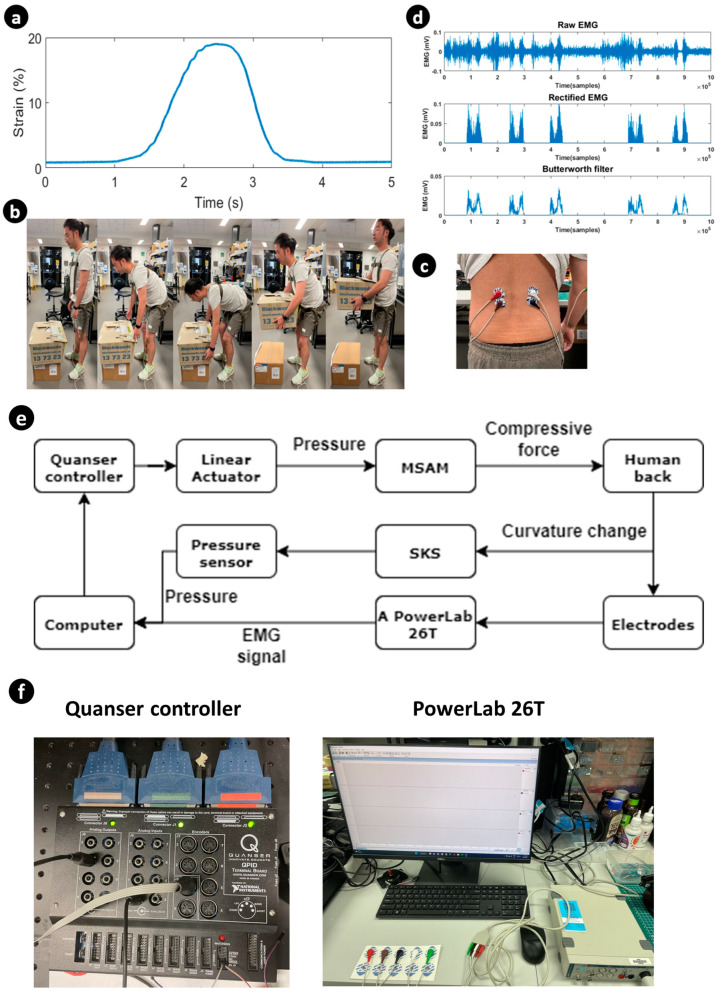
Outline of the experimental setup. (**a**) The pressure signal over one-time lifting is converted to a strain signal using the pressure–strain profile. (**b**) Demonstration of stoop lifting with the SARE, and the posture corresponding to the strain signal with time. (**c**) Demonstration of the electrodes’ position for LES. (**d**) EMG signals. (**e**) System connection for experiments. (**f**) Experimental equipment.

EMG aids in the detection and monitoring of the electrical activity of muscles. sEMG uses surface electrodes to detect multiple motor units above the skin. It provides a quantitative method to assess muscle function, movement patterns and localized muscle fatigue to inform clinical decision-making processes. A PowerLab 26T (AD Instruments, Sydney, Australia), which collects two signals simultaneously at a 10 kHz sampling rate, is used for data acquisition. Pre-gelled conductive adhesive recording electrodes are used on the skin surface. Because the erector spinae and latissimus dorsi muscles have many overlapped places, the electrodes are placed 5 cm laterally from the median line of the third lumbar vertebra for detecting LES signals, above the erector spinae and tendons of the latissimus dorsi muscle. Tendons themselves do not generate electrical activity like muscles do. Therefore, it reduces the influence of the latissimus dorsi muscle on the experiment results. Ground electrodes are placed on the distal upper arm (Figure 7c).

For the connection of the system, a computer is connected to a Quanser controller (QUANSER, Court Markham, ON, Canada), pressure sensor (Honeywell, Charlotte, NC, USA) and PowerLab 26T (Figure 7e,f). The pressure sensor detects the strain and curvature of the human back and sends data to the computer in real time. The computer sends electrical signals to the Quanser controller, based on data from the pressure sensor. Then, the Quanser controller controls the MSAM to assist the human back. The Power-Lan 26T generated the EMG signal and analysed it in MATLAB 2023.

#### 3.1.2. Data Collection

The male volunteers (N = 4, height = 182 cm ± 4.1 and weight = 67.4 kg ± 8.9) of this experiment were recruited based on the absence of a history of back pain and being in good health. The experiment was conducted under the UNSW Ethics Approval No. HC210717. Before the experiment, the volunteers were instructed to relax their spines for 30 min. All four experiments were completed with stoop lifting, whereby the volunteers were required to maintain a straight lower lumbar and flex at the hip joint. A 5 kg weight was positioned 5 cm in front of the volunteer’s toe. Upon touching the bottom of the weight after stooping, the volunteers returned to an erect standing posture, thereby maintaining a consistent bending angle of the back. The data were collected for four scenarios. In the first scenario, the subject mimicked stoop lifting without any load. The second scenario involved the subject carrying a load, and both scenarios were initially performed without any assistance. Subsequently, the experiments were repeated with the assistance of the SARE, and the EMG data were recorded from all four scenarios for further analysis (Figure 7b).

#### 3.1.3. Data Processing

The EMG signal processing was divided into four parts: bandpass filter, rectification, smoothing and integration. A bandpass filter (50–400 Hz) was applied through digital processing in the PowerLab 26T data acquisition system. Smoothing was achieved in MATLAB with the Butterworth function (10 Hz), designed to reduce the shape of the frequency response. Figure 7d shows the rectified and smoothing process steps of a raw EMG signal recorded from the LES. After filtering the EMG signal, the iEMG signal was calculated by integrating the area under the curve of the rectified EMG signal. The efficiency of the assist device is calculated as

(8) $$E=1-\frac{EMG_{assist}}{EMG_{no-assist}}$$

$EMG_{assist}$ and $EMG_{no-assist}$ are the iEMG signal when the volunteer tests with/without the SARE.

### 3.2. Sitting Posture Monitoring

**Figure 8:**
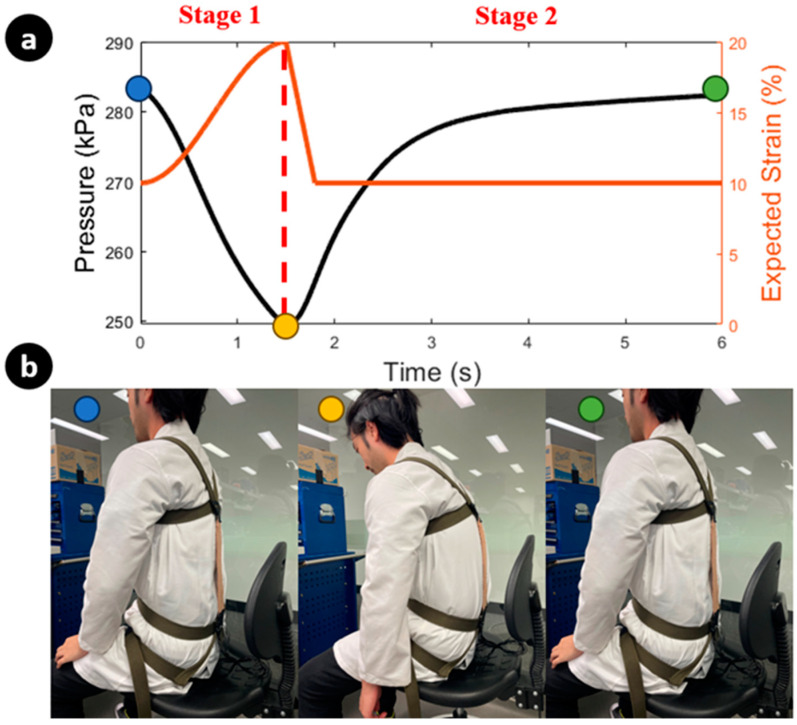
Overview of monitoring flexed posture. (**a**) The left y-axis of the figure illustrates the inner pressure change in the SKS, while the right y-axis depicts the expected strain of the MSAM under linear actuator control during posture correction. (**b**) Demonstration of posture correction with the SARE, along with the corresponding strain signal over time, reflecting changes in posture. Each colour represents the corresponding posture and inner pressure of the SKS.

As a demonstration, one SKS and MSAM are used for monitoring a flexed sitting posture. The experimental overview of monitoring a flexed posture is depicted in Figure 8, consisting of two stages. In stage 1, the posture changes from an erect to a flexed position, and then from a flexed back to erect in stage 2. During the first transition, the elongation of the SKS increases due to the increased strain on the back, leading to a drop in the inner pressure of the SKS (indicated by the black line). This is accompanied by the expected strain of the MSAM elongating with the SKS (indicated by the orange line) through the control box. Once the inner pressure of the SKS reaches a specific threshold of 250 kPa, the linear actuator initiates a withdrawal of pressure through the control box, marking the commencement of stage 2 (indicated by the red line). In this stage, the MSAM begins to apply assistive force to rectify the sitting posture and the expected strain suddenly drops. The inner pressure of the SKS returns to its original value, consistent with an erect posture. Figure 8a shows that the pressure signal varies under an actual stoop lifting scenario.

## 4. Results

**Figure 9:**
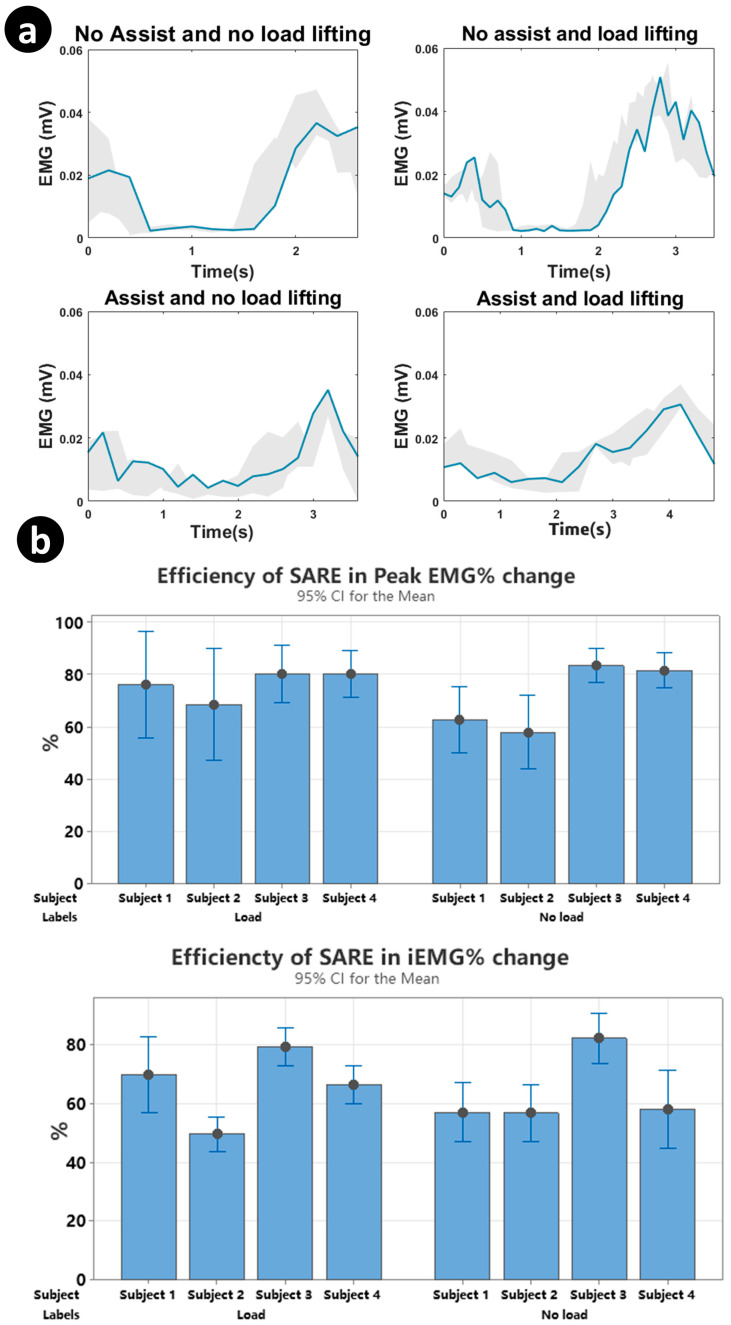
Results of processed EMG signal for four experiments. (**a**) Overview of the entire lifting period with a combination of all valid tasks for different lifting types. (**b**) Column plot of the efficiency of the SARE at peak EMG signals and iEMG signals for four subjects with error bars.

From Figure 9a, the shaded region is the trajectory of the root mean square (RMS) EMG signal of each experiment. The decreased and flattened RMS EMG amplitudes demonstrate a decreased muscular activity when the volunteer wears the SARE. The mean iEMG signals are reduced when the volunteer wears the SARE, both with and without load. According to Equation (8), the efficiencies of wearing the SARE are calculated and demonstrated in Figure 9b. Through wearing the SARE during stoop lifting, the peak EMG signals of the lumbar erector spinae are reduced by 22.8% ± 12 for lifting 5 kg weights and 27.1% ± 14 in empty-handed conditions. Moreover, the iEMG decreased by 34.7% ± 11.8 for lifting 5 kg weights and 36% ± 13.3 in empty-handed conditions. This demonstrates that the SARE can assist the LES during repetitive lifting with and without load.

## 5. Discussion and Conclusions

In this paper, we introduced a new design of a lightweight, soft exosuit, capable of providing compression forces to assist in stoop lifting through the use of a MSAM. Constructed from soft microtubules and helical coils, the MSAM can elongate by 20% of its initial length upon receiving a hydraulic volume of 10 mL, applying 35 N over the entire 60 mm stroke range and reaching a maximum of 50 N at 20% strain. Additionally, we introduced a promising soft knitting sensor, demonstrating its potential as a flexible, stretchable tool for monitoring changes in spinal curvature and sitting posture. The sensor’s capabilities are further highlighted by a drop in pressure of 90 kPa as elongation reaches 20% strain under an initial inner pressure of 300 kPa within the SKS, exhibiting a sensitivity of 4.5 kPa per percentage strain.

A preliminary EMG experiment was conducted to evaluate the exosuit’s capacity to reduce iEMG and peak EMG. The exosuit conforms to the curvature of the human back with nearly infinite DOFs and remains parallel to the LES without impeding human movement. Additionally, the real-time monitoring of the back’s strain sensor facilitates precise actuator control. Various exosuits have been reported in the literature to reduce peak EMG activity during lifting tasks, with reduction percentages ranging from 6 to 48% [53]. The SARE has achieved a promising 32% reduction in peak EMG activity for loaded lifting tasks. While [53] focused on rigid exoskeletons, it is more appropriate to compare the SARE with other soft exosuits. For example, a soft, textile-based, cable-driven exosuit demonstrated a 16% reduction in the peak EMG of the back extensor [54], and a TSA-based soft exosuit reported a 21.4–25.2% reduction [23]. Additionally, a study involving a cable-driven continuum soft exosuit reported a 30% reduction in the compression and shear force of the L5-S1. Overall, the SARE’s performance demonstrates a promising prospect for reducing muscular workload during lifting tasks.

Furthermore, the versatility of our smart textiles enabled the integration of multiple SKSs and MSAMs into the wearable device for additional functions. We demonstrated the sitting-posture-monitoring function. By employing multiple SKS sensors, the system can detect movements in various directions, enhancing its ability to identify improper postures and potentially correct them with the MSAM, addressing the issue where the monitoring function is currently restricted to observing only the sagittal plane and the curvature of the lumbar spine, as well as the relative motion of the smart textile against the body. This enhances the system to not only detect these specific aspects, but also to broaden its scope to include a more comprehensive analysis of various postural alignments. Therefore, the system will have the ability to correct sitting postures more effectively, recognizing and adjusting even subtle deviations, thereby providing a more robust and versatile solution for maintaining proper body alignment.

The next stage of system development will focus on optimizing the MSAM-driven smart textiles through improvements to the wire diameter, spring rate, spring materials, silicone tube materials and high-intensity guide tubes to be capable of holding higher hydraulic pressures. The addition of multiple layers of MSAMs on the exosuit is also being considered to generate greater compression force. Ultimately, the goal is for this exosuit is to facilitate stoop lifting entirely. One area in which this system can be improved in the future is in the implementation of nonlinear modelling and adaptive control, which can significantly enhance the system’s performance during the lifting process. Adding EMG signals and machine learning techniques to predict the bending motion to intelligently assist the user when needed is also recommended. Finally, conducting more user studies would demonstrate the effectiveness and benefits of the system for use in practice.

In conclusion, we have unveiled a novel design for a lightweight, soft exosuit that incorporates MSAMs to provide assistive compression forces to aid in stoop lifting. By integrating SKSs into wearable devices, we can monitor the strain of human movement, with MSAMs being activated by the SKSs. We have also showcased the multifunctional capabilities of the SKS and MSAM combination for both monitoring and correcting sitting postures. These innovations hold promising potential applications in fields such as rehabilitation, sports training and occupational health, where addressing and preventing back-related injuries is of utmost importance. Through this development, we anticipate contributing to the advancement of the robotic research community and foresee the potential to positively impact society.

## Data Availability

Data is unavailable due to privacy or ethical restrictions.

## List of Abbreviations

Abbreviation	Meaning
WMSDs	Work-related musculoskeletal disorders
SARE	Spine assistance robotic exosuit
EMG	Electromyography
iEMG	Integrated electromyogram
sEMG	Surface electromyography
LBP	Lower back pain
IMU	Inertial measurement unit
ADL	Activities of daily living
SKS	Soft knitting sensor
LES	Lumbar erector spinae
LD	Latissimus dorsi
TSA	Twisted string actuator
MSAMs	Multi-soft artificial muscles
FE	Finite element
DOF	Degree of freedom
RMS	Root mean square
HAL	Hybrid assistive limb
